# Longitudinal association of hypertension and dyslipidemia with cognitive function in community-dwelling older adults: the SONIC study

**DOI:** 10.1038/s41440-023-01271-5

**Published:** 2023-04-24

**Authors:** Yuko Nakamura, Mai Kabayama, Kayo Godai, Winston Tseng, Hiroshi Akasaka, Koichi Yamamoto, Yoichi Takami, Yasushi Takeya, Yasuyuki Gondo, Saori Yasumoto, Madoka Ogawa, Ayaka Kasuga, Yukie Masui, Kazunori Ikebe, Yasumichi Arai, Tatsuro Ishizaki, Hiromi Rakugi, Kei Kamide

**Affiliations:** 1 Division of Health Sciences, Osaka University, Graduate School of Medicine, 1-7 Yamadaoka, Suita, Osaka, 567-0871 Japan; 2 School of Public Health, University of California, Berkeley, 2199 Addison Street Room 50, Berkeley, CA 94720-7358 USA; 3 Department of Geriatric and General Medicine, Osaka University, Graduate School of Medicine, 2-2 Yamadaoka, Suita, Osaka, 567-0871 Japan; 4 Department of Clinical Thanatology and Geriatric Behavioral Sciences, Osaka University, Graduate School of Human Sciences, 1-2 Yamadaoka, Suita, Osaka, 567-0871 Japan; 5 Research Team for Human Care, Tokyo Metropolitan Institute of Gerontology, 35-2 Sakae-cho, Itabashi-ku, Tokyo, 173-0015 Japan; 6 Center for Super Centenarian Medical Research, Keio University School of Medicine, 35 Shinanomachi, Shinjuku-ku, Tokyo, 160-8582 Japan

**Keywords:** Cognitive functioning, Dyslipidemia, Hypertension, Older population

## Abstract

The associations among cognitive function, hypertension, and dyslipidemia in older adults are controversial. Therefore, we investigated the associations among cognitive decline, hypertension, dyslipidemia, and their combination in community-dwelling older people in their 70s, 80s, and 90s in the long-term observational Septuagenarians, Octogenarians, Nonagenarians, Investigation with Centenarians (SONIC) study. We administered the Montreal Cognitive Assessment Japanese version (MoCA-J) by trained geriatricians and psychologists, and conducted blood testing and blood pressure (BP) measuring by medical staff involving 1186 participants. We performed multiple regression analysis to assess the relationships among hypertension, dyslipidemia, their combination, and lipid and BP levels with cognitive function at the 3-year follow-up after adjusting for covariate factors. At the baseline, the percentage of the combination of hypertension and dyslipidemia was 46.6% (*n* = 553), hypertension was 25.6% (*n* = 304), dyslipidemia was 15.0% (*n* = 178), and that without hypertension or dyslipidemia was 12.7% (*n* = 151). Conducting multiple regression analysis, no significant correlation was found between the combination of hypertension and dyslipidemia and MoCA-J score. In the group with the combination, high high-density lipoprotein cholesterol (HDL) levels resulted in higher MoCA-J scores at the follow-up (β = 0.06; *P* < 0.05) and high diastolic BP (DBP) also resulted in higher MoCA-J scores (β = 0.08; *P* < 0.05). The results suggest that high HDL and DBP levels of individuals with HT & DL and high SBP levels of individuals with HT were associated with cognitive function in community-dwelling older adults.

**Figure:**
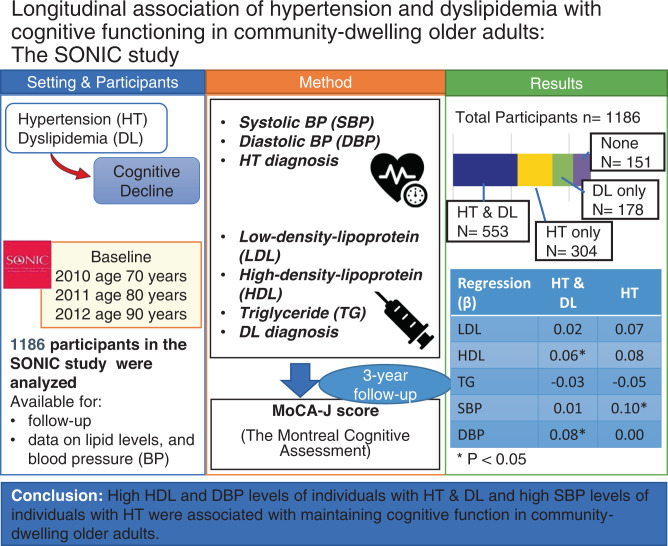
In the SONIC study, which is an epidemiological study of Japanese older persons aged 70 years or older, a disease-specific examination suggested that high HDL and DBP levels of individuals with hypertension & dyslipidemia and high SBP levels of individuals with hypertension were associated with maintaining cognitive function in community-dwelling older adults

In the SONIC study, which is an epidemiological study of Japanese older persons aged 70 years or older, a disease-specific examination suggested that high HDL and DBP levels of individuals with hypertension & dyslipidemia and high SBP levels of individuals with hypertension were associated with maintaining cognitive function in community-dwelling older adults

## Abstract (web summary)

In the SONIC study, which is an epidemiological study of Japanese older persons aged 70 years or older, a disease-specific examination suggested that high HDL and DBP levels of individuals with hypertension & dyslipidemia and high SBP levels of individuals with hypertension were associated with maintaining cognitive function in community-dwelling older adults

## Introduction

As the population ages, there is an urgent need to focus on how to deal with cognitive dysfunction. Cognitive dysfunction is a significant medical challenge, especially in developed countries, and is one of the critical diseases that reduce the quality of life and the activity of daily living (ADL) of the older adults [1]. In addition, cognitive decline is a concern even if it does not lead to dementia, and preventing cognitive decline is a significant medical issue [2].

Vascular contributions to cognitive impairment and dementia in later life are common, and hypertension is a major risk factor for cognitive impairment [3]. It has been reported that 1 billion individuals worldwide have hypertension [4]. Hypertension is associated with changes in the vascular structure and function, leading to cognitive decline [5]. However, the findings from some studies that have examined the association between later-life hypertension and cognitive function are inconsistent. Some studies failed to find an association [5–8]. On the other hand, several studies reported a U-shaped or linear association between blood pressure (BP) and cognitive function [9–11]. In a longitudinal investigation, our research group showed that hypertension and diabetes mellitus are associated with cognitive decline in older adults in their 70s, although no association was found between hypertension and cognitive function [12].

Dyslipidemia is also one of the main lifestyle-related diseases which causes atherosclerosis of the vascular system, especially ischemic cerebrovascular disease [13, 14], which is a risk for Alzheimer’s disease. Some previous studies showed that midlife lipid levels such as total cholesterol (TCHO), low-density lipoprotein cholesterol (LDL-C), high-density lipoprotein cholesterol (HDL-C) and triglycerides (TG) had affected on cognitive decline in old age [15]. In older populations, on the other hand, prospective studies showed that high lipid levels in old age are mostly unrelated or actually protect the cognitive function [16–20].

In such older populations, hypertension and dyslipidemia are conditions that can coexist frequently. The National Health and Nutrition Examination Survey (NHANES III) showed that 64% of patients with hypertension also have dyslipidemia and, conversely, approximately 47% of patients with dyslipidemia have hypertension [21]. However, few studies have examined the prospective effects of the combination of hypertension and dyslipidemia on the cognitive function of older adults. It is an urgent issue to clarify the relationship between blood pressure and lipid control and geriatric syndromes, including dementia, in Japan and East Asia, where the population is aging rapidly. Therefore, our goal was to consider the 3-year longitudinal effect of hypertension, dyslipidemia, their combination, and other risk factors on cognitive decline in individuals at 70~90 years of age using data from the Septuagenarians, Octogenarians and Nonagenarians Investigation with Centenarians (SONIC) study which is a longitudinal cohort study targeting older ages, overall and within selected subgroups.

Point of view

- Clinical relevance
  High high-density lipoprotein cholesterol (HDL-C) and diastolic blood pressure (DBP) levels of individuals with hypertension & dyslipidemia and high systolic blood pressure (SBP) levels of individuals with hypertension were associated with maintaining cognitive function.
- Future direction:
  A long-term study to examine the effect of blood pressure and lipid levels on cognitive function is warranted.
- Consideration for the Asian population
  While hypertension and dyslipidemia affect the vascular structure and function related to cognitive function, the Asian population has a higher hypertension ratio than other regions.

## Method

### Study population

The prospective study was part of the SONIC study, an ongoing prospective cohort study targeting community-dwelling older people in their 70s, 80s, 90s, or older in two regions: eastern and western areas of Japan. A detailed description of the study design and protocol has been published elsewhere [22]. We used a narrow age-range cohort design, with each cohort including individuals whose ages fell within a 3-year range.

**Fig. 1:**
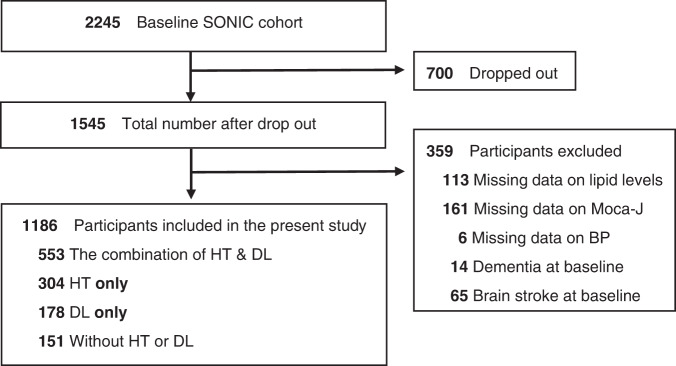
Flow chart of the study participants. HT hypertension, DL dyslipidemia, MoCA-J Japanese version of Montreal Cognitive Assessment, BP blood pressure

This study used data from the baseline of older people in their 70s, 80s, and 90s in 2010, 2011, and 2012, respectively, and the follow-up survey after 3 years. All participants provided written informed consent. The study was approved by the Institutional Review Board of Osaka University Graduate School of Medicine, Dentistry and Human Science and the Tokyo Metropolitan Geriatric Hospital and Institute of Gerontology (Tokyo, Japan; approval number 266, H22-9, 22 018 and 38, respectively). We excluded participants who did not join the follow-up survey after 3 years and who had missing data on BP, lipid levels, and cognitive function at the baseline. Those with a history of dementia or stroke at the baseline were also excluded. The final analytical sample included 1,186 participants for the current analysis (Fig. 1). We also analyzed after excluding the participants who had cerebra and cardiovascular diseases during the follow-up period. Data are shown as Supplementary Material.

### Measurements

#### BP measurement and classification

A physician or trained nurse, using a mercury sphygmomanometer, obtained BP measurements from the left and right arms of the patients while they sat after at least 1 min of rest, and the means of the 2 measurements of both arms were used in the analysis. According to the criteria of the Japanese Society of Hypertension guidelines for the management of hypertension (JSH 2014), the diagnosis of hypertension was based on BP values over 140/90 mm Hg and the use of antihypertensive treatment at the time of the first contact [23].

#### Lipid measurement and classification

The lipid levels were assessed by casual blood testing performed by doctors or nurses. The variables used in the analysis were LDL-C, HDL-C, and TG. LDL-C was calculated using the Friedewald formula [24]:

$${{{{{{{\mathrm{LDL}}}}}}}} - {{{{{{{\mathrm{C}}}}}}}} = {{{{{{{\mathrm{TCHO}}}}}}}}-{{{{{{{\mathrm{HDL}}}}}}}} \mbox{-} {{{{{{{\mathrm{C}}}}}}}}-{{{{{{{\mathrm{TG}}}}}}}}/{{{{{{{\mathrm{5}}}}}}}}$$

According to the criteria of the Japan Atherosclerosis Society (JAS 2017), the diagnosis of dyslipidemia was based on LDL ≥ 140 mg dL, HDL < 40 mg dL, TG ≥ 150 mg dL, or the use of antidyslipidemia treatment at the time of the first contact [25].

### Assessment of cognitive functioning

We used the Japanese version of the Montreal Cognitive Assessment (MoCA-J) as a general index of the cognitive status. MoCA is a brief cognitive screening tool for detecting mild cognitive impairment in older adults. MoCA consists of a 1-page 30-point test administered by trained geriatricians and psychologists, with higher scores reflecting more favorable cognitive functioning. MoCA assesses the following domains of cognition: visuospatial abilities (3 points), naming task (3 points), attention task (6 points), language (3 points), abstraction task (2 points), delayed recall (5 points), and orientation (6 points). MoCA-J showed a favorable reliability and better validity for predicting mild cognitive impairment than conventional cognitive tests. If the participants’ educational level was 12 years or less, we added 1 point to the total MoCA score.

We used the MoCA-J total score as a predictor of cognitive functioning.

### Statistical analyses

At the baseline and follow-up examination, descriptive data were summarized and presented as the mean ± s.d. values and proportions. Multiple linear regression models were produced for each group to calculate the standardized regression coefficients (β) expressing independent associations between the variables.

First, we conducted regression including total participants to analyze each factor affecting cognitive function in all the subjects with model 1. When examining the group with hypertension, the group with dyslipidemia, and the group with the combination of hypertension and dyslipidemia as independent variables, the group without hypertension and dyslipidemia was used as a reference. The covariates of model 1 included age, sex, diabetes, body mass index (BMI), smoking and drinking history, and the MoCA-J total score at the baseline.

Then, we selected participants with each disease and performed multiple regression analyses, respectively. The group with the combination of hypertension and dyslipidemia was analyzed in model 2, the group with hypertension only was analyzed in model 3, the group with dyslipidemia only was analyzed in model 4, and the group without hypertension or dyslipidemia was analyzed in model 1.

The covariates of model 2 included age, sex, diabetes, BMI, smoking and drinking history, anti-hypertensive treatment, anti-dyslipidemia treatment, and the MoCA-J total score at the baseline.

The covariates of model 3 included age, sex, diabetes, BMI, smoking and drinking history, anti-hypertensive treatment, and the MoCA-J total score at the baseline.

The covariates of model 4 included age, sex, diabetes, BMI, smoking and drinking history, anti-dyslipidemia treatment, and the MoCA-J total score at the baseline.

All statistical analyses were performed using SPSS Statistics 22 (IBM Japan, Tokyo, Japan). All reported *P*-values are two-tailed, and *P* < 0.05 was considered to be significant.

## Results

### Characteristics of participants

Between 2010 and 2012, 2245 participants in the SONIC study were considered eligible. Of those, 700 participants dropped out before the follow-up survey, and 359 participants were excluded due to missing data for each potential confounding factor, having dementia, or a history of brain stroke at the baseline. Finally, 1186 participants were included in the present study (Fig. 1).

The characteristics of participants at the baseline and follow-up are shown in Table 1. At the baseline, 80-year-old participants were predominant in the combined hypertension and dyslipidemia group and the hypertension group, while 70-year-old participants were predominant in the dyslipidemia group and without hypertension & dyslipidemia group. More than half of the participants with hypertension were taking medication for hypertension. The group with hypertension and dyslipidemia was dominated by participants with TG ≥ 150, and the dyslipidemia group was dominated by participants with LDL ≥ 140. In the group with dyslipidemia and the group without hypertension or dyslipidemia, mean MoCA-J scores were higher than for the total participants.

**Table 1:** Characteristics of the study population at the baseline

	Total *n* = 1186	With HT&DL *n* = 553	With HT *n* = 304	With DL *n* = 178	Without HT&DL *n* = 151
*Baseline*					
Male (%)	567 (47.8)	247 (44.7)	174 (57.2)	63 (35.4)	83 (55.0)
Age					
70 yr (%)	573 (48.3)	235 (42.5)	125 (41.1)	117 (65.7)	96 (63.6)
80 yr (%)	551 (46.5)	286 (51.7)	160 (52.6)	54 (30.3)	51 (33.8)
90 yr (%)	62 (5.2)	32 (5.8)	19 (6.3)	7 (3.9)	4 (2.6)
SBP (mmHg)	142.4 ± 19.2	149.2 ± 17.0	149.3 ± 16.3	125.8 ± 12.5	123.3 ± 12.9
DBP (mmHg)	78.1 ± 10.7	80.1 ± 10.8	80.5 ± 10.5	73.2 ± 8.4	72.1 ± 9.0
HT (%)	857 (72.3)	553 (100.0)	304 (100.0)	0 (0)	0 (0)
Medication for HT (%)	544 (45.9)	361 (65.3)	183 (60.2)	0 (0)	0 (0)
LDL-C (mg dL-1)	119.9 ± 29.8	123.1 ± 33.3	111.2 ± 20.4	134.1 ± 30.8	109.4 ± 21.1
LDL-C ≥ 140 (%)	279 (23.5)	192 (34.7)	0 (0)	87 (48.9)	0 (0)
HDL-C (mg dL-1)	61.0 ± 15.9	57.4 ± 15.3	64.7 ± 14.6	60.9 ± 16.9	66.6 ± 15.9
HDL-C < 40 (%)	84 (7.1)	63 (11.4)	0 (0)	21 (11.8)	0 (0)
TG (mg dL-1)	132.0 ± 73.6	163.2 ± 80.0	92.4 ± 30.5	144.8 ± 78.1	82.1 ± 27.1
TG ≥ 150 (%)	345 (29.1)	278 (50.3)	0 (0)	67 (37.6)	0 (0)
DL (%)	731 (61.6)	553 (100.0)	0 (0)	178 (100.0)	0 (0)
Medication for DL (%)	295 (24.9)	234 (42.3)	0 (0)	61 (34.3)	0 (0)
DM (%)	122 (10.3)	69 (2.5)	27 (8.9)	17 (9.6)	9 (6.0)
BMI (kg m-2)	22.7 ± 3.3	23.3 ± 3.0	22.5 ± 3.9	22.1 ± 2.6	21.1 ± 2.8
MoCA-J total score (0–30)	22.9 ± 3.5	22.8 ± 3.4	22.5 ± 3.7	23.5 ± 3.6	23.4 ± 3.1
Smoking history (%)	426 (35.9)	198 (35.8)	116 (38.2)	58 (32.6)	54 (35.8)
Drinking history (%)	493 (41.6)	212 (38.3)	162 (53.3)	49 (27.5)	70 (46.4)
*Follow-up*					
SBP (mmHg)	138.6 ± 18.3	141.5 ± 17.6	143.5 ± 7.9	130.3 ± 16.2	128.2 ± 17.0
DBP (mmHg)	75.5 ± 11.1	76.1 ± 11.1	76.7 ± 11.6	74.0 ± 10.3	72.8 ± 10.2
HT (%)	830 (70.0)	461 (83.4)	257 (84.5)	67 (37.6)	45 (29.8)
Medication for HT (%)	559 (47.1)	345 (62.4)	177 (58.2)	21 (11.8)	16 (10.6)
LDL-C (mg dL-1)	114.9 ± 29.9	116.2 ± 32.5	110.4 ± 24.1	124.5 ± 32.1	107.7 ± 24.1
LDL-C ≥ 140 (%)	210 (17.7)	112 (20.3)	29 (9.5)	57 (32.0)	12 (7.9)
HDL-C (mg dL-1)	59.7 ± 15.3	57.2 ± 15.0	62.7 ± 14.9	59.6 ± 15.7	63.0 ± 15.2
HDL-C < 40 (%)	85 (7.2)	57 (10.3)	9 (3.0)	15 (8.4)	4 (2.6)
TG (mg dL-1)	133.9 ± 76.7	155.3 ± 82.1	107.3 ± 62.0	141.7 ± 74.3	100.7 ± 56.3
TG ≥ 150 (%)	364 (30.7)	235 (42.5)	52 (17.1)	57 (32.0)	20 (13.2)
DL (%)	720 (60.7)	438 (79.2)	104 (34.2)	136 (76.4)	42 (27.8)
Medication for DL (%)	335 (28.2)	238 (43.0)	26 (8.6)	61 (34.3)	10 (6.6)
Combinated HT (%)	530 (0.4)	370 (0.7)	91 (0.3)	26 (0.2)	19 (0.1)
DM (%)	161 (13.6)	91 (16.5)	35 (11.5)	19 (10.7)	16 (10.6)
BMI (kg m-2)	22.7 ± 3.1	23.3 ± 3.2	22.4 ± 3.2	22.3 ± 2.7	21.2 ± 2.8
MoCA-J total score (0–30)	23.0 ± 3.9	23.1 ± 3.7	22.4 ± 4.3	23.6 ± 3.9	23.4 ± 3.4
Smoking history (%)	437 (36.8)	201 (36.3)	118 (38.8)	60 (33.7)	58 (38.4)
Drinking history (%)	486 (41.0)	213 (38.5)	152 (50.0)	52 (29.2)	69 (45.7)
Cerebro & Cardio vascular disease (%)	33 (2.8)	23 (4.2)	4 (1.3)	4 (2.2)	2 (1.3)

*HT* hypertension, *DL* dyslipidemia, *SBP* systolic blood pressure, *DBP* diastolic blood pressure, *LDL-C* low-density lipoprotein-cholesterol, *HDL-C* high-density lipoprotein-cholesterol, *TG* triglycerides, *DM* diabetes mellitus, *BMI* body mass index, *MoCA-J* the Japanese version of Montreal Cognitive Assessment

Systolic BP (SBP) and diastolic BP (DBP) at the follow-up were lower than at the baseline in the hypertension and dyslipidemia combined and hypertension groups. In contrast, SBP and DBP increased at the follow-up in the dyslipidemia and without hypertension and dyslipidemia group. LDL-C and HDL-C were decreased in all groups compared with the baseline level, but TG was increased only in the hypertensive group and without hypertension and dyslipidemia group.

The characteristics of the participants at the baseline who didn’t have brain or heart diseases during the follow-up period is shown in Supplementary Table 1.

### Multiple regression analysis

Table 2 shows the standardized univariate coefficients and multi-regression coefficients as predictors of the MoCA-J total score at the time of the follow-up. In the hypertension and dyslipidemia group, a high HDL-C level and high DBP at the baseline were correlated significantly with the MoCA-J total score at the time of follow-up after adjusting for age, sex, diabetes, BMI, smoking and drinking history, anti-hypertensive treatment, anti-dyslipidemia treatment, and the MoCA-J total score at the baseline in model 2 (β = 0.06; *P* < 0.05) (β = 0.08; *P* < 0.05). In the hypertension group, SBP at the baseline was correlated significantly with MoCA-J in model 3 (β = 0.10; *P* < 0.05), whereas TG at the baseline was not a significant predictor (model 3).

**Table 2:** Standardized multi-regression coefficients (β) as predictors of MoCA-J total score at follow-up

		Total *n* = 1186	With HT&DL *n* = 553	With HT *n* = 304	With DL *n* = 178	Without HT&DL *n* = 151
		Model 1	Model 2	Model 3	Model 4	Model 1
HT & DL	Univariate coefficients	−0.05				
	Multi-regression coefficients (β)	0.04				
HT	Univariate coefficients	−0.11**				
	Multi-regression coefficients (β)	−0.02				
DL	Univariate coefficients	0.02				
	Multi-regression coefficients (β)	0.01				
LDL	Univariate coefficients	0.06*	−0.03	0.15**	0.10	0.11
	Multi-regression coefficients (β)	0.04	0.02	0.07	0.01	0.07
HDL	Univariate coefficients	0.13***	0.14***	0.15**	0.18*	0.09
	Multi-regression coefficients (β)	0.04	0.06*	0.08	0.01	0.00
TG	Univariate coefficients	−0.01	−0.02	−0.02	−0.08	−0.05
	Multi-regression coefficients (β)	−0.01	−0.03	−0.05	0.04	0.00
SBP	Univariate coefficients	−0.05	−0.05	0.09	−0.05	−0.03
	Multi-regression coefficients (β)	0.04	0.01	0.10*	0.03	0.03
DBP	Univariate coefficients	0.06*	0.11*	0.12*	0.08	−0.02
	Multi-regression coefficients (β)	0.04	0.08*	0.00	0.03	0.03

The covariates of model 1 include age, sex, diabetes, BMI, smoking and drinking history, and the MoCA-J total score at the baseline

The covariates of model 2 include model 1 + anti-hypertensive treatment and anti-dyslipidemia treatment

The covariates of model 3 include model 1 + anti-hypertensive treatment

The covariates of model 4 include model 1 + anti-dyslipidemia treatment

Parameter estimates (β) can be interpreted as differences in MoCA-J total scores for each 1 mg dL increase in LDL, HDL, and TG, and 1 mmHg increase in SBP and DBP

*HT* hypertension, *DL* dyslipidemia, *LDL-C* low-density lipoprotein-cholesterol, *HDL-C* high-density lipoprotein-cholesterol, *TG* triglycerides, *SBP* systolic blood pressure, *DBP* diastolic blood pressure, *MoCA-J* the Japanese version of Montreal Cognitive Assessment

*P < 0.05, **P < 0.01, ***P < 0.001

Table 3 shows the standardized univariate coefficients and multi-regression coefficients as predictors of the change in MoCA-J total score between the baseline and follow-up. In the hypertension and dyslipidemia group, HDL and DBP at the baseline were significant predictors of the change of MoCA-J in model 2 (β = 0.08; *P* < 0.05) (β = 0.10; *P* < 0.05). In the hypertension group, SBP was a significant predictor of the change of MoCA-J in model 3 (β = 0.12; *P* < 0.05). These results were similar when brain or heart diseases during the follow-up period were adjusted as a covariate factor (Supplementary Tables 2, 3).

**Table 3:** Standardized multi-regression coefficients (β) as predictors of change of MoCA-J total score between baseline and follow-up

		Total *n* = 1186	With HT&DL *n* = 553	With HT *n* = 304	With DL *n* = 178	Without HT&DL *n* = 151
		Model 1	Model 2	Model 3	Model 4	Model 1
HT & DL	Univariate coefficients	0.04				
	Multi-regression coefficients (β)	0.05				
HT	Univariate coefficients	−0.02				
	Multi-regression coefficients (β)	−0.02				
DL	Univariate coefficients	0.01				
	Multi-regression coefficients (β)	0.02				
LDL	Univariate coefficients	0.04	0.03	0.03	0.03	0.08
	Multi-regression coefficients (β)	0.05	0.03	0.09	0.01	0.08
HDL	Univariate coefficients	0.02	0.08	0.05	−0.03	−0.07
	Multi-regression coefficients (β)	0.05	0.08*	0.09	0.01	0.00
TG	Univariate coefficients	−0.01	−0.06	−0.08	0.06	0.04
	Multi-regression coefficients (β)	−0.01	−0.04	−0.05	0.05	0.00
SBP	Univariate coefficients	0.05	0.00	0.13*	0.05	0.03
	Multi-regression coefficients (β)	0.05	0.02	0.12*	0.04	0.04
DBP	Univariate coefficients	0.05	0.11**	0.00	0.02	−0.01
	Multi-regression coefficients (β)	0.05	0.10*	−0.01	0.03	0.03

The covariates of model 1 include age, sex, diabetes, BMI, smoking and drinking history, and the MoCA-J total score at the baseline

The covariates of model 2 include model 1 + anti-hypertensive treatment and anti-dyslipidemia treatment

The covariates of model 3 include model 1 + anti-hypertensive treatment

The covariates of model 4 include model 1 + anti-dyslipidemia treatment

Parameter estimates (β) can be interpreted as differences in MoCA-J total scores for each 1 mg dL increase in LDL, HDL, and TG, and 1 mmHg increase in SBP and DBP

*HT* hypertension, *DL* dyslipidemia, *LDL-C* low-density lipoprotein-cholesterol, *HDL-C* high-density lipoprotein-cholesterol, *TG* triglycerides, *SBP* systolic blood pressure, *DBP* diastolic blood pressure, *MoCA-J* the Japanese version of Montreal Cognitive Assessment

*P < 0.05, **P < 0.01

Supplementary Tables 4, and 5 show the multi-regression analysis of the participants who didn’t have brain or heart diseases during the follow-up period. Likewise, a high HDL and a DBP at baseline were associated with a high Moca-J score in the hypertension and dyslipidemia group, even when subjects with brain or heart disease during the follow-up period were excluded.

## Discussion

A high BP and high lipid level in middle age have been suggested to be predictive indicators of cognitive functioning and dementia [15], whereas the association between hypertension or dyslipidemia and cognitive functioning has been controversial in older age groups [16–20]. We aimed to clarify the association between BP or lipids and cognitive functioning in a general Japanese older population using the SONIC study, a large-scale longitudinal cohort study of older Japanese individuals in the general population. In addition, the combination of hypertension and dyslipidemia is common among older adults; however, few studies have examined the risk factor of cognitive decline in participants with hypertension and dyslipidemia. Therefore, we investigated a cohort study involving general Japanese older residents to specify predictors for each disease group. This approach in a longitudinal study may clarify differences in the influence of diseases on the association between cognitive functioning and BP or lipids among older local residents.

In our longitudinal analysis of 1186 subjects, we found that higher HDL-C level was associated with a higher score of MoCA-J in the hypertension and dyslipidemia group but not in the hypertension group, dyslipidemia group and the without hypertension and dyslipidemia group. We also found associations between higher DBP and a higher score of MoCA-J in the hypertension and dyslipidemia group, and between higher SBP and a higher score of MoCA-J in the hypertension group.

The causal role of lipids and BP in different types of dementia has been studied. According to the lipid level, several previous studies suggested that low HDL-C, low LDL-C, and low T-CHO were associated with Alzheimer’s disease around the age of 70 years old [16–18, 26–29]. Some meta-analyses showed no association or a weak association between lipid levels in later life and cognitive decline [16, 17, 19]. Several studies have investigated the relationship between BP in later life and cognitive function. One of the previous longitudinal studies on the relationship between BP and cognitive function suggested that lower SBP and DBP were associated with a higher risk of Alzheimer’s disease or cognitive impairment [30, 31]. Our study supported these findings of the protective effects of high HDL-C, SBP, and DBP on cognitive function. However, some longitudinal studies showed a different result from our study. One of the previous longitudinal studies examined the relationship between hypertension and dementia, and suggested that a higher SBP developed 4 years before the onset of Alzheimer’s disease in community-dwelling people under 76 years old [32]. Several previous studies using longitudinal observation performed a longer follow-up and included subjects with higher SBP values at the baseline than in our study; therefore, the results of SBP in the present study might not be concordant with those of previous studies [9–11, 32]. Our previous study examining the effects of SBP on cognitive function found a significant association between higher SBP and lower cognitive function among 70-year-olds, while among 90-year-olds, the opposite was found [33]. The present study analyzed subjects aged 70 to 90 together, and especially in the group with hypertension, a higher percentage was in their 80s and 90s compared to the other groups. This might have affected our present result that there was a relationship between a higher SBP and a higher MoCA-J score only in the group with hypertension. Another reason for the discrepancy with other studies involves the characteristics of participants in the SONIC study, whereas the participants in this study may be healthier than those in previous studies because most are community dwellers, and they were able to come to the investigation site by themselves. For such healthy participants, high BP in later life may have protected the cognitive function by properly transporting blood to the brain, rather than being a risk for cognitive decline. Some observational studies reported that hypertension is not related to increased dementia risk and AD at very advanced ages [34]. Corrada et al. reported a reduced risk of dementia in people aged ≥90 years with hypertension who reported the onset of hypertension after the age of 80 year [35]. Our previous cross-sectional study from SONIC study similarly revealed that SBP ≥ 140 mmHg in subjects with hypertension taking antihypertensive medication might be protective against declining cognitive function in age 90 group [33]. In older adults, especially at age 90, adequate cerebral perfusion may help to maintain normal cognition. Corrada et al. suggested that hypertension is a physiological compensatory mechanism to maintain adequate cerebral perfusion in the face of age-associated vascular changes [35]. Kitagawa et al. reported that individuals with lower cerebral blood flow have been found to have higher rates of cognitive decline [36]. This might be because of a sufficient nutritional supply to fuel the brain’s high metabolic activity [37]. Therefore, we suppose that higher SBP may be protective against declining cognitive function in older adults.

Interestingly, high HDL and high DBP at the baseline were correlated with the MoCA-J total score at the time of follow-up in community-dwelling older adults aged 70 or older in the hypertension and dyslipidemia group, as shown in Table 2. These findings are novel. From these results, we suggest that maintaining high HDL and DBP may have a protective influence on future cognitive function in populations with the combination of hypertension and dyslipidemia compared with hypertension or dyslipidemia only.

Dyslipidemia and hypertension are well-known risk factors for vascular reformation such as atherosclerosis which causes ischemic heart diseases, but the effect of dyslipidemia on the brain has not yet been convincingly demonstrated. For example, low T-CHO or LDL-C was associated with the onset of ischemic stroke [38]. In addition, cholesterol is an essential nutrient for the brain, and plays a crucial role in the development and maintenance of neuronal function [39]. Although most cholesterol in the brain is synthesized in the central nervous system and not carried from the plasma into the brain because of the blood-brain barrier [19], oxysterols are able to pass through the blood-brain barrier, and over 90% of circulating hydroxycholesterol originates in the brain. As a consequence, blood concentrations of hydroxycholesterol reflect central nerve system cholesterol turnover [40]. Previous studies showed that high cholesterol and hydroxycholesterol levels have some effect on memory consolidation in experimental studies, and suggested that hydroxycholesterol could link peripheral cholesterol to the cognitive function [41]. Other reports indicate that low HDL is involved in the amyloid-β plaques causing Alzheimer’s disease or cognitive impairment [42]. As these studies suggest, HDL may be indirectly linked to the cholesterol in the brain. In addition, previous studies showed that carotid artery atherosclerosis was an independent risk factor for the development of cognitive impairment [43, 44]. HDL plays an important role in the prevention of atherosclerosis [45] and in this study, low HDL might have indicated the progress of atherosclerosis. This could also affect the current result. Some epidemiological studies found a relationship between lower HDL levels and cognitive impairment in older individuals [16–18, 26–29, 46], which directly supports our findings. The study found that low HDL is also a risk for cognitive decline in older Japanese adults with hypertension and dyslipidemia, a novel finding.

According to hypertension, the association of BP in later life and the oldest old with cognitive function is unclear, with both harmful and protective effects of high BP on cognition [5–9]. Our study results support this beneficial effect on the cognitive function. Hypertension affects the vascular structure and function; it causes atherosclerosis and is associated with changes in the structure of the vascular wall, and chronic hypertension exerts marked effects on cerebrovascular function, disrupting major factors regulating cerebral circulation [47]. DBP tends to spontaneously decrease with aging as a result of stiffening of the arterial wall and decreasing vessel wall compliance [48], and low DBP is an indicator of atherosclerosis in the older individuals. Like the low HDL values, atherosclerosis may have progressed in the participants with low DBP values. Recent findings showed that the prevalence of hypertension did not differ between those who are cognitively normal and those with mild cognitive impairment (MCI), suggesting that hypertension-related cognitive impairment involves skills other than memory [49]. In addition, there are some reports that vascular risk factors influence executive functioning to a greater extent and are not necessarily related to memory [47]. Our study used MoCA-J as a predictor of cognitive decline. MoCA-J assesses the visuospatial ability, naming task, attention task, language, abstraction task, delayed recall, and orientation. We used the MoCA-J total score as the outcome and did not analyze MoCA-J sub-scores. This may have affected the current result. In general, cognitive impairment progresses over decades. Thus, it might not be easy to identify the association during this short follow-up time.

The key strength of our SONIC study was that we investigated a cohort study involving community-dwelling participants of old and oldest ages to specify predictors under conditions involving an older Japanese population. Dividing the subjects into those with combined hypertension and dyslipidemia, hypertension, dyslipidemia, and other subjects allows us to consider the impact of risk by disease. To our knowledge, this disease-specific approach at older ages in longitudinal studies has the potential to clarify the impact of hypertension, dyslipidemia, their combination, BP and lipid levels in later life on cognitive function. In addition, the MoCA-J score was sensitive compared with Mini Mental State Examination; therefore, we identified additional outcomes to examine the longitudinal association between hypertension and cognitive functioning.

Our study had several limitations. We conducted only venue blood pressure data, which could have led to measurement error and an underestimation of the effect on cognitive function. Additionally, the follow-up rate in our study was low (1545/2245) because there were several expected reasons for dropout from the follow-up survey, such as the following: they had no time to participate in the study, they moved out of the area we surveyed, or they could not participate in the follow-up study because of poor health conditions. We also likely missed a few cases in the participants who died or were institutionalized in nursing homes before the follow-up survey. This situation would tend to bias our results toward underestimating the influence of hypertension and dyslipidemia on cognitive functioning. Finally, blood samples were obtained without fasting. Measured serum lipid levels may be influenced by the post-prandial state if the interval until blood sampling after a meal is very short.

In conclusion, the present study resulted that high HDL and DBP levels of individuals with HT & DL and high SBP levels of individuals with HT were associated with cognitive function in community-dwelling older adults after a 3-year follow-up.

Therefore, we recommend that low DBP and HDL levels may be noted in the older population over 70 years of age, especially with the combination of hypertension and dyslipidemia from the point of view of preventing cognitive decline.

### Perspective of Asia

The aging of the population is progressing in Asia, particularly in East Asia, including Japan. Most studies examining the relationship between hypertension, dyslipidemia, and cognitive function in older adults have been conducted on Caucasians, but few have been conducted on Asians. In addition, hypertension is more common in Japan than in other regions, and in many cases, hypertension and dyslipidemia are combined. This study of community-dwelling older Japanese will provide new insights into the management of hypertension and dyslipidemia to prevent dementia in the older population in Asia.

## Conclusion

The present study resulted that high HDL and DBP levels of individuals with HT & DL and high SBP levels of individuals with HT were associated with cognitive function in community-dwelling older adults after a 3-year follow-up.

Therefore, we recommend that low DBP and HDL levels may be noted in the older population over 70 years of age, especially with the combination of hypertension and dyslipidemia from the point of view of preventing cognitive decline.

## Supplementary information

Supplementary Table 1

Supplementary Table 2

Supplementary Table 3

Supplementary Table 4

Supplementary Table 5

## References

[1] Ferri CP, Prince M, Brayne C, Brodaty H, Fratiglioni L, Ganguli M (2005). Global prevalence of dementia: a Delphi consensus study. Lancet.

[2] Sperling RA, Aisen PS, Beckett LA, Bennett DA, Craft S, Fagan AM (2011). Toward defining the preclinical stages of Alzheimer’s disease: Recommendations from the National Institute on Aging‐Alzheimer’s Association workgroups on diagnostic guidelines for Alzheimer’s disease. Alzheimers Dement.

[3] Gorelick PB, Scuteri A, Black SE, Decarli C, Greenberg SM, Iadecola C (2011). Vascular contributions to cognitive impairment and dementia. Stroke.

[4] Mozaffarian D, Benjamin EJ, Go AS, Arnett DK, Blaha MJ, Cushman M (2015). Heart disease and stroke statistics—2015 update. Circulation..

[5] Hebert LE, Scherr PA, Bennett DA, Bienias JL, Wilson RS, Morris MC (2004). Blood pressure and late-life cognitive function change: a biracial longitudinal population study. Neurology.

[6] Johnson KC, Margolis KL, Espeland MA, Colenda CC, Fillit H, Manson JE (2008). A prospective study of the effect of hypertension and baseline blood pressure on cognitive decline and dementia in postmenopausal women: the Women’s Health Initiative Memory Study. J Am Geriatr Soc.

[7] Solfrizzi V, Panza F, Colacicco AM, D’Introno A, Capurso C, Torres F (2004). Vascular risk factors, incidence of MCI, and rates of progression to dementia. Neurology.

[8] Yaffe K, Haan M, Blackwell T, Cherkasova E, Whitmer RA, West N (2007). Metabolic syndrome and cognitive decline in elderly Latinos: findings from the Sacramento Area Latino Study of Aging study. J Am Geriatr Soc.

[9] Bohannon AD, Fillenbaum GG, Pieper CF, Hanlon JT, Blazer DG (2002). Relationship of race/ethnicity and blood pressure to change in cognitive function. J Am Geriatr Soc.

[10] Dregan A, Stewart R, Gulliford MC (2013). Cardiovascular risk factors and cognitive decline in adults aged 50 and over: a population-based cohort study. Age Ageing.

[11] Yasar S, Ko JY, Nothelle S, Mielke MM, Carlson MC (2011). Evaluation of the effect of systolic blood pressure and pulse pressure on cognitive function: the Women’s Health and Aging Study II. PLoS One.

[12] Ryuno H, Kamide K, Gondo Y, Kabayama M, Oguro R, Nakama C (2017). Longitudinal association of hypertension and diabetes mellitus with cognitive functioning in a general 70-year-old population: the SONIC study. Hypertens Res.

[13] Barter P, Gotto AM, LaRosa JC, Maroni J, Szarek M, Grundy SM (2007). HDL cholesterol, very low levels of LDL cholesterol, and cardiovascular events. N. Engl J Med.

[14] Reitz C, Tang MX, Schupf N, Manly JJ, Mayeux R, Luchsinger JA (2010). A summary risk score for the prediction of Alzheimer disease in elderly persons. Arch Neurol.

[15] Power MC, Rawlings A, Sharrett AR, Bandeen‐Roche K, Coresh J, Ballantyne CM (2018). Association of midlife lipids with 20‐year cognitive change: A cohort study. Alzheimers Dement.

[16] Anstey KJ, Ashby-Mitchell K, Peters R (2017). Updating the evidence on the association between serum cholesterol and risk of late-life dementia: review and meta-analysis. J Alzheimers Dis.

[17] Anstey KJ, Lipnicki DM, Low LF (2008). Cholesterol as a risk factor for dementia and cognitive decline: a systematic review of prospective studies with meta-analysis. Am J Geriatr Psychiatry.

[18] Benito-León J, Vega-Quiroga S, Villarejo-Galende A, Bermejo-Pareja F (2015). Hypercholesterolemia in elders is associated with slower cognitive decline: a prospective, population-based study (NEDICES). J Neurol Sci.

[19] Reitz C (2013). Dyslipidemia and the risk of Alzheimer’s disease. Curr Atheroscler Rep.

[20] Reitz C, Luchsinger J, Tang M-X, Manly J, Mayeux R (2005). Impact of plasma lipids and time on memory performance in healthy elderly without dementia. Neurology.

[21] Kalra S, Kalra B, Agrawal N (2010). Combination therapy in hypertension: an update. Diabetol Metab Syndr.

[22] SONIC Study. A Longitudinal Cohort Study of the Older People as Part of a Centenarian Study [press release]. In Pachana NA (ed.), Encyclopedia of Geropsychology, Springer Science+Business Media Singapore, Encyclopedia of Geropsychology 2017.

[23] Shimamoto K, Ando K, Fujita T, Hasebe N, Higaki J, Horiuchi M (2014). The Japanese Society of Hypertension guidelines for the management of hypertension (JSH 2014). Hypertens Res.

[24] Friedewald WT, Levy RI, Fredrickson DS (1972). Estimation of the concentration of low-density lipoprotein cholesterol in plasma, without use of the preparative ultracentrifuge. Clin Chem.

[25] Kinoshita M, Yokote K, Arai H, Iida M, Ishigaki Y, Ishibashi S (2018). Japan Atherosclerosis Society (JAS) guidelines for prevention of atherosclerotic cardiovascular diseases 2017. J Atheroscler Thromb.

[26] Demeester N, Castro G, Desrumaux C, De Geitere C, Fruchart JC, Santens P (2000). Characterization and functional studies of lipoproteins, lipid transfer proteins, and lecithin:cholesterol acyltransferase in CSF of normal individuals and patients with Alzheimer’s disease. J Lipid Res.

[27] Merched A, Xia Y, Visvikis S, Serot JM, Siest G (2000). Decreased high-density lipoprotein cholesterol and serum apolipoprotein AI concentrations are highly correlated with the severity of Alzheimer’s disease. Neurobiol Aging.

[28] Reitz C, Tang M-X, Luchsinger J, Mayeux R (2004). Relation of plasma lipids to Alzheimer disease and vascular dementia. Arch Neurol.

[29] van den Kommer TN, Dik MG, Comijs HC, Fassbender K, Lütjohann D, Jonker C (2009). Total cholesterol and oxysterols: early markers for cognitive decline in elderly?. Neurobiol Aging.

[30] Sabayan B, Oleksik AM, Maier AB, van Buchem MA, Poortvliet RK, de Ruijter W (2012). High blood pressure and resilience to physical and cognitive decline in the oldest old: the Leiden 85-plus Study. J Am Geriatr Soc.

[31] Verghese J, Lipton RB, Hall CB, Kuslansky G, Katz MJ (2003). Low blood pressure and the risk of dementia in very old individuals. Neurology..

[32] Li G, Rhew IC, Shofer JB, Kukull WA, Breitner JC, Peskind E (2007). Age-varying association between blood pressure and risk of dementia in those aged 65 and older: a community-based prospective cohort study. J Am Geriatr Soc.

[33] Kabayama M, Kamide K, Gondo Y, Masui Y, Nakagawa T, Ogawa M (2020). The association of blood pressure with physical frailty and cognitive function in community-dwelling septuagenarians, octogenarians, and nonagenarians: the SONIC study. Hypertens Res.

[34] Qiu C, Winblad B, Fratiglioni L (2005). The age-dependent relation of blood pressure to cognitive function and dementia. Lancet Neurol.

[35] Corrada MM, Hayden KM, Paganini-Hill A, Bullain SS, DeMoss J, Aguirre C (2017). Age of onset of hypertension and risk of dementia in the oldest-old: The 90+ Study. Alzheimers Dement.

[36] Kitagawa K, Oku N, Kimura Y, Yagita Y, Sakaguchi M, Hatazawa J (2009). Relationship between cerebral blood flow and later cognitive decline in hypertensive patients with cerebral small vessel disease. Hypertens Res.

[37] Cunnane S, Nugent S, Roy M, Courchesne-Loyer A, Croteau E, Tremblay S (2011). Brain fuel metabolism, aging, and Alzheimer’s disease. Nutrition.

[38] Ryglewicz D, Rodo M, Kunicki PK, Bednarska-Makaruk M, Graban A, Lojkowska W (2002). Plasma antioxidant activity and vascular dementia. J Neurol Sci.

[39] Pfrieger FW (2003). Cholesterol homeostasis and function in neurons of the central nervous system. Cell Mol Life Sci.

[40] Papassotiropoulos A, Lütjohann D, Bagli M, Locatelli S, Jessen F, Rao ML (2000). Plasma 24S-hydroxycholesterol: a peripheral indicator of neuronal degeneration and potential state marker for Alzheimer’s disease. Neuroreport.

[41] Björkhem I, Cedazo-Minguez A, Leoni V, Meaney S (2009). Oxysterols and neurodegenerative diseases. Mol Asp Med.

[42] Agarwal M, Khan S (2020). Plasma lipids as biomarkers for Alzheimer’s disease: a systematic review. Cureus.

[43] Johnston SC, O’Meara ES, Manolio TA, Lefkowitz D, O’Leary DH, Goldstein S (2004). Cognitive impairment and decline are associated with carotid artery disease in patients without clinically evident cerebrovascular disease. Ann Intern Med.

[44] Newman AB, Fitzpatrick AL, Lopez O, Jackson S, Lyketsos C, Jagust W (2005). Dementia and Alzheimer’s disease incidence in relationship to cardiovascular disease in the Cardiovascular Health Study cohort. J Am Geriatr Soc.

[45] Parhofer KG (2015). Increasing HDL-cholesterol and prevention of atherosclerosis: a critical perspective. Atheroscler Suppl.

[46] Raffaitin C, Féart C, Le Goff M, Amieva H, Helmer C, Akbaraly TN (2011). Metabolic syndrome and cognitive decline in French elders: the three-city study. Neurology.

[47] Iadecola C, Yaffe K, Biller J, Bratzke LC, Faraci FM, Gorelick PB (2016). Impact of hypertension on cognitive function: a scientific statement from the American Heart Association. Hypertension.

[48] Duprez DA (2008). Systolic hypertension in the elderly: addressing an unmet need. Am J Med.

[49] Peltz CB, Corrada MM, Berlau DJ, Kawas CH (2012). Cognitive impairment in nondemented oldest‐old: Prevalence and relationship to cardiovascular risk factors. Alzheimer’s Dement.

