# Supplementary material for: Longitudinal association of hypertension and dyslipidemia with cognitive function in community-dwelling older adults: the SONIC study
Source: Hypertens Res. 2023 Apr 24;46(8):1829–39. doi: 10.1038/s41440-023-01271-5 (PMC10404512; doi:10.1038/s41440-023-01271-5)
Supplement: Supplementary file 1 — Supplementary Table 1 [file 41440_2023_1271_MOESM1_ESM.doc]

**Supplementary t**able 1 Characteristics of the study population at the baseline

|  | Total  N=1153 | With HT&DL N=530 | With HT N=300 | With DL N=174 | Without HT&DL N=149 |
| --- | --- | --- | --- | --- | --- |
| ***Baseline*** | | | | | |
| Male(%) | 551 (47.8) | 236 (44.5) | 170 (56.7) | 63 (36.2) | 82 (55.0) |
| Age 　70 yr (%) | 552 (47.9) | 223 (42.1) | 121 (40.3) | 114 (65.5) | 94 (63.1) |
| 80 yr (%) | 540 (46.8) | 276 (52.1) | 160 (53.3) | 53 (30.5) | 51 (34.2) |
| 90 yr (%) | 61 (5.3) | 31 (5.8) | 19 (6.3) | 7 (4.0) | 4 (2.7) |
| SBP (mmHg) | 142.5 ± 19.2 | 149.4 ± 16.8 | 149.5 ± 16.2 | 125.8 ± 12.6 | 123.2 ± 12.9 |
| DBP (mmHg) | 78.2 ± 10.7 | 80.3 ± 10.8 | 80.5 ± 10.4 | 73.1 ± 8.4 | 72.1 ± 9.1 |
| HT (%) | 830 (72.0) | 530 (100) | 300 (100) | 0 (0) | 0 (0) |
| Medication for HT (%) | 521 (45.2) | 342 (64.5) | 179 (59.7) | 0 (0) | 0 (0) |
| LDL-C (mg dL-1) | 120.2 (29.6) | 123.7 (33.3) | 111.3 (20.2) | 133.8 (30.2) | 109.4 (21.0) |
| LDL-C≥140 (%) | 273 (23.7) | 188 (35.5) | 0 (0) | 85 (48.9) | 0 (0) |
| HDL-C (mg dL-1) | 61.2 ± 15.8 | 57.7 ± 15.2 | 64.8 ± 14.6 | 60.8 ± 16.8 | 66.7 ± 16.0 |
| HDL-C<40 (%) | 76 (6.6) | 55 (10.4) | 0 (0) | 21 (12.1) | 0 (0) |
| TG (mg dL-1) | 131.5 ± 73.8 | 163.2 ± 80.5 | 92.2 ± 30.5 | 145.3 ± 78.8 | 81.9 ± 27.1 |
| TG≥150 (%) | 329 (28.5) | 263 (49.6) | 0 (0) | 66 (37.9) | 0 (0) |
| DL (%) | 704 (61.1) | 530 (100) | 0 (0) | 174 (100) | 0 (0) |
| Medication for DL (%) | 279 (24.2) | 221 (41.7) | 0 (0) | 58 (33.3) | 0 (0) |
| DM (%) | 119 (10.3) | 66 (12.5) | 27 (9.0) | 49 (28.2) | 9 (6.0) |
| BMI (kg m-2) | 22.6 ± 3.3 | 23.3 ± 3.0 | 22.5 ± 3.9 | 22.1 ± 2.6 | 21.1 ± 2.8 |
| MoCA-J total score (0–30) | 22.9 ± 3.5 | 22.8 ± 3.4 | 22.5 ± 3.7 | 23.5 ± 3.6 | 23.5 ± 3.1 |
| Smoking history (%) | 414 (35.9) | 192 (36.2) | 112 (37.3) | 56 (32.2) | 54 (36.2) |
| Drinking history (%) | 481 (41.7) | 205 (38.7) | 159 (53.0) | 48 (27.6) | 69 (46.3) |
| ***Follow-up*** | | | | | |
| SBP (mmHg) | 138.6 ± 18.4 | 141.5 ± 17.8 | 143.7 ± 17.8 | 130.2 ± 16.3 | 128.2 ± 17.1 |
| DBP (mmHg) | 75.6 ± 11.1 | 76.3 ± 11.1 | 76.8 ± 11.6 | 74.0 ± 10.4 | 72.8 ± 10.2 |
| HT (%) | 804 (69.7) | 441 (83.2) | 253 (84.3) | 66 (37.9) | 44 (29.5) |
| Medication for HT (%) | 535 (46.4) | 327 (61.7) | 173 (57.7) | 20 (11.5) | 15 (10.1) |
| LDL-C (mg dL-1) | 115.2 ± 29.8 | 116.8 ± 32.6 | 110.7 ± 24.0 | 124.4 ± 32.0 | 107.8 ± 24.1 |
| LDL-C≥140 (%) | 204 (17.7) | 108 (20.4) | 29 (9.7) | 55 (31.6) | 12 (8.1) |
| HDL-C (mg dL-1) | 59.9 ± 15.3 | 57.4 ± 14.9 | 62.8 ± 15.0 | 59.5 ± 15.7 | 63.0 ± 15.3 |
| HDL-C<40 (%) | 80 (6.9) | 52 (9.8) | 9 (3.0) | 15 (8.6) | 4 (2.7) |
| TG (mg dL-1) | 133.2 ± 76.6 | 154.5 ± 82.1 | 107.3 ± 62.2 | 141.5 ± 74.6 | 100.6 ± 56.4 |
| TG≥150 (%) | 349 (30.3) | 223 (42.1) | 51 (17.0) | 55 (31.6) | 20 (13.4) |
| DL (%) | 695 (60.3) | 418 (78.9) | 103 (34.3) | 132 (75.9) | 42 (28.2) |
| Medication for DL (%) | 323 (28.0) | 228 (43.0) | 26 (0.09) | 59 (33.9) | 10 (6.7) |
| Combinated HT (%) | 511 (44.3) | 353 (66.6) | 90 (30.0) | 49 (28.2) | 19 (12.8) |
| DM (%) | 152 (13.2) | 84 (15.8) | 33 (11.0) | 19 (10.9) | 16 (10.7) |
| BMI (kg m-2) | 22.6 ± 3.1 | 23.3 ± 3.1 | 22.5 ± 3.2 | 22.3 ± 2.7 | 21.1 ± 2.8 |
| MoCA-J total score (0–30) | 23.0 ± 3.9 | 23.0 ± 3.8 | 22.4 ± 4.3 | 23.6 ± 4.0 | 23.4 ± 3.3 |
| Smoking history (%) | 423 (36.7) | 193 (36.4) | 114 (38.0) | 58 (33.3) | 58 (38.9) |
| Drinking history (%) | 475 (41.2) | 208 (39.2) | 149 (49.7) | 50 (28.7) | 68 (45.6) |

Abbreviations: HT, hypertension; DL, dyslipidemia; SBP, systolic blood pressure; DBP, diastolic blood pressure; LDL-C, low-density lipoprotein-cholesterol; HDL-C, high-density lipoprotein-cholesterol; TG, triglycerides; DM, diabetes mellitus; BMI, body mass index; MoCA-J, the Japanese version of Montreal Cognitive Assessment.
