# Supplementary material for: Longitudinal association of hypertension and dyslipidemia with cognitive function in community-dwelling older adults: the SONIC study
Source: Hypertens Res. 2023 Apr 24;46(8):1829–39. doi: 10.1038/s41440-023-01271-5 (PMC10404512; doi:10.1038/s41440-023-01271-5)
Supplement: Supplementary file 2 — Supplementary Table 2 [file 41440_2023_1271_MOESM2_ESM.doc]

**Supplementary table 2 Standardized multi-regression coefficients (β) as predictors of MoCA-J total score at follow-**up

|  | | Total  n=1186 | With HT&DL  n=553 | With HT  n=304 | With DL  n=178 | Without HT&DL  n=151 |
| --- | --- | --- | --- | --- | --- | --- |
|  |  | Model 1 | Model 2 | Model 3 | Model 4 | Model 1 |
| HT & DL | ***Univariate coefficients*** | -0.05 |  |  |  |  |
|  | ***Multi-regression coefficients (β)*** | 0.03 |  |  |  |  |
| HT | ***Univariate coefficients*** | **-0.11**** |  |  |  |  |
|  | ***Multi-regression coefficients (β)*** | -0.02 |  |  |  |  |
| DL | ***Univariate coefficients*** | 0.02 |  |  |  |  |
|  | ***Multi-regression coefficients (β)*** | 0.01 |  |  |  |  |
| LDL | ***Univariate coefficients*** | **0.06*** | -0.03 | **0.15**** | 0.10 | 0.11 |
|  | ***Multi-regression coefficients (β)*** | 0.04 | 0.02 | 0.07 | -0.01 | 0.07 |
| HDL | ***Univariate coefficients*** | **0.13***** | **0.14***** | **0.15**** | **0.18*** | 0.09 |
|  | ***Multi-regression coefficients (β)*** | 0.04 | **0.07*** | 0.08 | 0.01 | -0.01 |
| TG | ***Univariate coefficients*** | -0.01 | -0.02 | -0.02 | -0.08 | -0.05 |
|  | ***Multi-regression coefficients (β)*** | -0.01 | -0.03 | -0.05 | 0.05 | 0.00 |
| SBP | ***Univariate coefficients*** | -0.05 | -0.05 | 0.09 | -0.05 | -0.03 |
|  | ***Multi-regression coefficients (β)*** | 0.04 | 0.02 | **0.10*** | 0.03 | 0.04 |
| DBP | ***Univariate coefficients*** | **0.06*** | **0.11*** | **0.12*** | 0.08 | -0.02 |
|  | ***Multi-regression coefficients (β)*** | 0.04 | **0.08*** | 0.00 | 0.02 | 0.03 |

Abbreviations: HT, hypertension; DL, dyslipidemia; LDL-C, low-density lipoprotein-cholesterol; HDL-C, high-density lipoprotein-cholesterol; TG, triglycerides; SBP, systolic blood pressure; DBP, diastolic blood pressure; MoCA-J, the Japanese version of Montreal Cognitive Assessment.

The covariates of model 1 include age, sex, diabetes, BMI, smoking and drinking history, the MoCA-J total score at the baseline, and Cerebro & Cardio vascular disease during follow-up.

The covariates of model 2 include model 1 + anti-hypertensive treatment and anti-dyslipidemia treatment.

The covariates of model 3 include model 1 + anti-hypertensive treatment.

The covariates of model 4 include model 1 + anti-dyslipidemia treatment.

Parameter estimates (β) can be interpreted as differences in MoCA-J total scores for each 1 mg dL increase in LDL, HDL, and TG, and 1mmHg increase in SBP and DBP.

*P<0.05, **P<0.01, ***P<0.001.
